# Editorial: Engineering Nucleic Acids-Based Functional Nanomaterials, Nanodrugs, and Biosensors

**DOI:** 10.3389/fbioe.2022.915229

**Published:** 2022-06-16

**Authors:** Jabrane Jouha, Fengli Li, Wen-Ta Su, Chenguang Fan, Dayong Yang, Hai Xiong

**Affiliations:** ^1^ Institute for Advanced Study, Shenzhen University, Shenzhen, China; ^2^ Department of Chemical Engineering and Biotechnology, National Taipei University of Technology, Taipei, Taiwan; ^3^ Department of Chemistry and Biochemistry, University of Arkansas, Fayetteville, AR, United States; ^4^ Key Laboratory of Systems Bioengineering (MOE), Frontiers Science Center for Synthetic Biology, School of Chemical Engineering and Technology, Tianjin University, Tianjin, China

**Keywords:** engineering DNA, ncRNA, cellular functions, biosensors, nanomaterials and nanodrugs

Recently, nucleic acid-based probes or functional nanomaterials have gained popularity for visualizing intracellular analytes due to their programmable nature, a property that has led to key advances in several areas of nanotechnology. Overall, these nanoplatforms can be used in diverse applications including, 1) intracellular imaging, which precisely images microRNAs, metal ions, gene damage, etc.; 2) biosensing, which utilizes functional nanomaterials (FNAs) for signal conversion and amplification and nanomaterials as a sensing platform to detect a wide range of targets; 3) biomedical, which includes early disease diagnosis, drug delivery, and tumor therapy. Furthermore, nucleic acid-based probes can be broadly classified into three groups: hybridization-based probes, aptamers, and DNAzymes. Meanwhile, research on the combination and functions of FNAs, such as DNA/RNA aptamers and ribozymes/DNAzymes, and nanomaterials *in vitro* and in cells, have been widely explored as molecular tools for various applications.

In the current topic, an overview of nucleic acids-based functional nanomaterials in pharmacology is provided through 9 articles by 49 authors, which contain 4 reviews and 5 original research papers.

One of the reviews summarized the recent progress in the design of DNA-based nanomaterials for nucleic acid delivery. In addition, the classification of DNA nanomaterials is categorized according to the components, e.g., pure DNA nanomaterials, DNA-inorganic hybrid nanomaterials, and DNA-organic hybrid nanomaterials were investigated in detail (Figure 1). Then, extensive applications of DNA nanomaterials especially in cancer therapy are given (Lv et al.).

**FIGURE 1:**
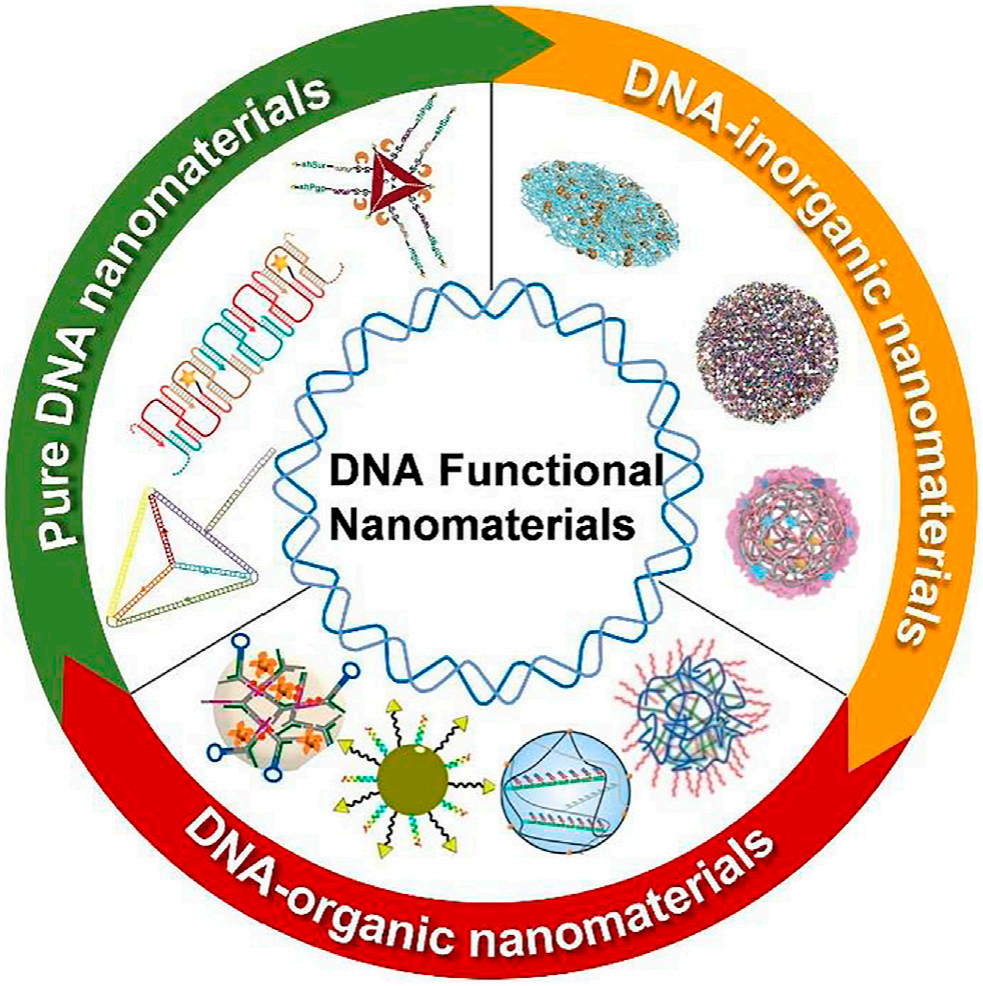
Schematic illustration of DNA functional nanomaterials for nucleic acid-based drug delivery. DNA functional nanomaterials were developed into three major categories. Reprinted with permission from ref. (Lv et al.). Copyright 2021 Frontiers.

As research on the structural and functional group features of FNAs and nanomaterials rapidly advances, many laboratories have reported numerous methods to design FNA-based nanomaterials to achieve a large number of special applications such as bioimaging, biosensing, phototherapy, and stimulation response delivery in living systems (Jouha and Xiong, 2021; Xu et al., 2019; Lake et al., 2019).

The special topic aims to present original studies covering critical aspects of FNA nanomaterials to address application issues in cancer therapy. Among them, Liu and colleagues reported a new nanomaterial based on extracellular vehicles (EVs), which were abundant with ncRNAs, containing chrysin and analyzed cell apoptosis in tongue squamous cell carcinoma (TSCC) cells, which is the most malignant tumor in the head and neck. Gold nanoparticles were carried by EVs-Chrysin (Au-EVs) to improve the therapeutic effect. By utilizing near-infrared radiation (NIR), Au-EVs have effectively enhanced cell apoptosis and significantly inhibited tumor growth *in vivo*. Moreover, Let-7a-3p was screened by RNA-seq in EVs-Chrysin and the overexpression of let-7a-3p induced cell apoptosis, which confirms the role of miRNAs in this assay (Yang et al.). In addition to gold nanoparticles, reduced graphene oxide (rGO) also played a vital role in developing DNA-based biosensors. As an intracellular carrier and quencher, rGO demonstrated its application for the highly sensitive detection and simultaneous *in situ* imaging of metal ions in living cells. For instance, Xiong et al. reported, a novel fluorescent probe employing the G-quadruplex form with consecutive pyrene modifications (_G_DNA-11) based on rGO to detect thrombin. Furthermore, using an optical or electrical G-quadruplex rGO-biosensor to recognize cancer cells provides the option for diagnostics and other biomedical applications (Zhao et al., 2022).

Meanwhile, another new type of nanotubes was successfully prepared by polyethylene glycol (PEG) coprecipitation to provide targeted delivery of paclitaxel (PTX). Because their shape favors long circulation, preferential distribution, and increased cellular uptake, particles of pepper mild mottle virus (PMMoV) are extended and may be useful as drug carriers. FA@PMMoV@PTX nanotubes were designed to selectively target tumor cells and to release the encapsulated PTX in an acidic microenvironment enabling a pH response. When FA@PMMoV@PTX nanotubes were incubated with tumor cells, they showed efficient cell uptake, and FA@PMMoV@PTX nanotubes displayed better cytotoxicity to free PTX, as indicated by cell survival and apoptosis of MCF-7 cells. This system is a great candidate for use in creating improved strategies for the targeted treatment of tumors (Peng et al.).

Another interesting application of DNA-Based nanosystem, a lysosomal proton-activatable DNA-based nanosystem was created to overcome the anti-CRISPR/Cas9 and DNAzyme impact of Mn^2+^ and so achieve efficient co-delivery of CRISPR/Cas9 and DNAzyme for combined gene therapy of breast cancer. By programming multi-functional sequences in one ultra-long ssDNA chain Ultra-long ssDNA chains, which contained the recognition of sgRNA in Cas9/sgRNA, DNAzyme sequence, and HhaI enzyme cleavage site and using it as the scaffold to regulate gene expressions to achieve a high therapeutic efficacy of tumors (Li et al., 2022).

Overall, the current Research Topic discussed the fundamental research and clinical applications of diverse nucleic acid-based probes and functional nanomaterials. In such an emerging field, engineering nucleic acids as functional nanomaterials provided an insightful undertaking to perform in the fields of nanomaterials and nanodrugs, and as biomarkers or biomedical imaging to detect biomolecules *in vivo* or *in vitro*.

## Important Note

All contributions to this Research Topic must be within the scope of the section and journal to which they are submitted, as defined in their mission statements. Frontiers reserves the right to guide an out-of-scope manuscript to a more suitable section or journal at any stage of peer review.
